# Hospital of Oral Surgery

**Published:** 1887-08

**Authors:** Robert S. Ivy


### HOSPITAL OF ORAL SURGERY, PHILADELPHIA.

### CLINIC OF PROFESSOR GARRETSON.

REPORTED BY ROBERT S. IVY, D. D. S.

### MUCOID ENGORGEMENT OF THE ANTRUM.

The patient, a lady, complained of fullness of the cheek, neuralgic pains, and tenderness of the roof of the mouth on the left side. Roots of diseased teeth, molars or bicuspids, which pierce the sinus, from contiguity of the peridental membrane and the mucous lining of the cavity, are frequent causes of such trouble as the lady complained of. Examination of the mouth, however, excluded such cause in this case, as it revealed an unbroken alveolar ridge from the cuspid tooth to the tuberosity, all the teeth having been extracted some years previously. Attention was then directed to the natural opening from the sinus into the nares, which was found to be closed, the patient complaining of constant dryness of the nostril, readily accounted for by the lack of mucous discharge from the antrum, caused by the closure of the orifice. She also stated that an opening had been made into the bone twelve months previously, near the canine tooth, when she was suffering from a similar trouble. Inferring from this that the opening then made had become closed, a bistoury was thrust through the wall of the cavity in the region of the canine fossa, resulting in a muco-purulent discharge, the feeling of distention being immediately relieved. Through the opening thus formed the cavity was thoroughly cleansed by injections of dilute phenol-sodique, the treatment being continued daily for one week, the opening being meanwhile retained with a tent of cotton.

Remarking on the frequency of antral disease, the professor said that when of strictly local origin it is easily treated. When caused by a diseased tooth, with an abscess discharging into the sinus, the indication is to extract the tooth, thus forming a fistula by which the pus or other secretion can be removed, and at the same time an opening by which stimulating injections can be made. Such injections consist of phenol, carbolized water, or dilute tincture of iodine. Where the trouble has been allowed to continue, and the secretion has caused the lining membrane of the cavity to break down and ulcerate, injections of chloride of zinc, combined with one dram zinc sulphate and one pint of water, have proved very efficient.

A common cause of engorged antrum is catarrh of the Schneiderian membrane. A person takes cold and the inflammation of the lining membrane of the nares, by relation of continuity, extends into the sinus, which becomes congested to such an extent as to close the outlet. Under such circumstances the patient complains of a sense of heaviness in the cheek, attended by soreness of a dull, sluggish character. A mode of treatment often satisfactory, when the trouble is in its early stages, is to administer a saline cathartic, or better still to break up a limited congestion, give the patient at bed time one-sixth or one-quarter of a grain of sulphate of morphia dissolved in an ounce of liquor ammonia acetatis. Where the disease has advanced to engorgement, it is generally found necessary to open, as in the case under notice, or to extract the first or second molar, preferably the second, and to penetrate the sinus through the alveolus of the palatine root.

### LINGUAL EPITHELIOMA.

Mr. A., a gentleman sent to the clinic from California, upon examination presented expressions characteristic of epithelioma of the tongue, the disease involving two-thirds of the left side. In appearance the growth was of a purplish red color, having associated with it the giant granulations and indurated edges so frequently met with in epithelioma of a cancerous nature. The patient said he first noticed a slight soreness six months previously, apparently caused by ragged edges of some of his teeth. These were rounded off, but the abraded surface gradually enlarged, threatening to infiltrate the remainder of the tongue. Radical extirpation of the growth being desired, it was decided to remove a section of the organ, surrounding and including the growth, by the ecraseur. The first step was to distend the jaws, a screw-gag being placed between the teeth on the side opposite the seat of operation. A strong silk ligature was next passed through the anterior portion of the tongue, enabling an assistant to draw it forward into a more convenient position for operating. A bistoury was next thrust through the healthy tissue from the under side in the direction of the raphe, and this was followed by a piece of common annealed wire. A

second wire was placed slightly anterior to the first, and the ends of each attached to ecraseurs, the triangular section contained within the wires, when tense, included the diseased tissue. Pressure being put upon the handles of the instrument, the wires were tightened and the growth gradually severed from the surrounding tissue, the operation being completed by cutting away the small attached portion between the insertion of the wires. The time occupied in the retraction of the wires was about fifteen minutes, the hemorrhage by this means being reduced to a minimum. Owing to the extraordinary vitality of the tongue, the time occupied in recovering from such severe operations is short. In the case described, the patient was discharged from the hospital after eight days, no trace of the operation being perceptible.

